# Mid-term results of 150 TAVI comparing apical versus femoral approaches

**DOI:** 10.1186/s13019-015-0360-4

**Published:** 2015-11-03

**Authors:** Alain Rougé, Olivier Huttin, Rumas Aslam, Thibaud Vaugrenard, Thomas Jouve, Michael Angioi, Pablo Maureira

**Affiliations:** 1 Service de Chirurgie Cardiaque, CHU de Nancy, Hôpital Brabois, rue du Morvan, F-54511 Vandoeuvre-les-Nancy, France; 2 Service de Cardiologie, CHU de Nancy, Hôpital Brabois, rue du Morvan, F-54511 Vandoeuvre-les-Nancy, France; 3 Université de Lorraine, Nancy, France; 4 Service de Néphrologie - CHU Grenoble, Boulevard de la Chantourne, Grenoble, France

**Keywords:** TAVI: Transcatheter aortic-valve implantation, Aortic stenosis, VARC: Valve Academic Research Consortium

## Abstract

**Background:**

Transcatheter aortic-valve implantation (TAVI) is a new therapeutic choice for treating aortic stenosis in patients considered high risk for surgery. This blooming therapeutic technique still requires evaluation of medium and long term outcome.

**Method:**

We hereby report our results of the first 150 consecutive patients to receive TAVI implants in our population recruited from July 2009 to March 2013 in a retrospective and monocentric study. We analyzed long term morbidity and mortality criteria. We compared the apical and femoral approach results and researched predictors of cardiac mortality.

**Results:**

The mean monitoring period was 387.62 days, mean Euroscore was 21.8, and mean Society of Thoracic Surgeons (STS) risk score was 9.2. The success rate for the procedure was 94.6 %. A total of 39 patients died. The mortality rates at the immediate perioperative point, 30 days, 1 year, and 2 years, were 4 %, 11.3 %, 22.7 %, and 26 %, respectively. As regards complications, there were 10 hemodynamic complications (6.6 %) and 20 vascular (13.3 %), 11 cardiac tamponades (7.4 %), eight mechanical (5.3 %), ten major hemorrhagic (6.7 %), 14 pulmonary (9.3 %), and 18 infectious complications (12 %). When comparing the rates of reported complications in terms of different approaches, we observed significantly more hemodynamic complications in the apical group (*p* = 0.049). Pulmonary complications were also significantly more common in cases of apical approach (*p* = 0.029). The majority of the patients reported clear functional improvement throughout their follow-up.

**Conclusion:**

The results of the first 150 patients to receive the implant at the Nancy University Teaching Hospital (CHU Nancy) were consistent with findings in the literature. TAVI proved a credible and effective alternative to surgical valve replacement for patients at high risk during surgery.

## Background

Calcified aortic stenosis is the most common type of valvular heart disease found among adults in Western industrialized countries [1]. The etiology for this disease is mainly degenerative, therefore those affected are primarily of an advanced age. Observational studies demonstrate that its prevalence increases significantly with age, from 1.5 % for the 64–74-year age group to 4.8 % for those aged 75 and over [1]. Since the first percutaneous transcatheter implantation of an aortic valve prosthesis [2], transcatheter aortic-valve implantation (TAVI) has become a valid alternative to surgical aortic valve replacement [3–6]. Our study analyzed the first 150 patients treated with TAVI at the university hospital of Nancy (CHU Nancy) and compared the results according to approach method. We were particularly focused on studying and analyzing the mortality rates at 31 days, 1 year, and 2 years, along with the demographic features of the patient population, procedural information, complications, clinical follow-up, and echocardiography results.

## Methods

This study reports on the first 150 patients treated with TAVI at CHU Nancy from July 2009 to March 2013. This was a retrospective and monocentric study, yet does include prospective data gathered for the France 2 [5] and France TAVI registries.

Patients indicated for TAVI procedures were selected according to the guidelines of the Haute Autorité de Santé (HAS), the French national health authority, after the possibility of surgery was ruled out. Patient assessment was carried out at a multidisciplinary meeting, taking into account surgical risk scores (logistic Euroscore >20 % or Society of Thoracic Surgeons [STS] >10 %) and comorbidities. Each patient treated was fully informed and signed an informed consent form. The following information was collected before the procedure: demographic information, blood parameter values, and echocardiogram data. These same parameters were analyzed following surgery. The results were compared according to the type of approach used and the implantation success rate.

The effectiveness criteria consisted of the reduction in mean transaortic gradient and increase in aortic surface. The mortality rates were analyzed at several points: immediately perioperatively, at 30 days, 6 months, 1 year, 2 years, and 3 years. We also researched the predictive factors for cardiovascular mortality. Clinical follow-up, consisting of functional status according to the New York Heart Association [NYHA] classification, and echocardiography were carried out at the follow-up visits. Patient follow-up was performed at 6 months for all patients then at 1, 2, and even 3 years for the most elderly. This study involving the first 150 subjects thus extended over a period of 3 years and 7 months. We categorized the major complications in accordance with the Valve Academic Research Consortium (VARC) classification [7].

### Statistical analysis

The statistical analysis was carried out using the SPSS 17.0 software for Windows (Chicago, Illinois). Quantitative variables were compared using paired and non-paired t-tests or by analysis of variance (ANOVA). Qualitative variables were compared using chi-squared tests or Fisher’s exact test. We investigated the entire patient population for predictive event survival factors by means of univariate Cox regression model. In order to determine if these were the result of independent predictive factors, for each test a value of *p* < 0.05 was considered significant and for each significant variable we stated the relative risk and 95 % confidence interval (CI). The survival curves were determined using the Kaplan-Meier method.

## Results

The follow-up period extended from July 28^th^ 2009 to July 3^rd^ 2013, making a total follow-up period of 3 years and 9 months. In total, 150 patients were treated. The mean monitoring period was 387.62 days, with a median of 303.50 days (interquartile range [IQR]: 141.25-597.50).

The general features of the patient population have been presented in Table 1. Mean age at implantation was 82.6 years old. The mean Euroscore was 21.8 and mean STS was 9.2. All patients manifested symptoms. A study of the patient functional status revealed the following: 66 % exhibited Class III dyspnoea (NYHA); there was a high rate of coronary heart disease (46.7 %); both the femoral and apical approach groups were homogeneous.

**Table 1:** Patient characteristics at baseline

Characteristics	All patients (*n* = 150)	Transfemoral approach (*n* = 78)	Transapical approach (*n* = 72)	*p*
Male gender	68 (45.3 %)	35 (44.9 %)	33 (45.8 %)	NS
Age (years)	82.6 +/− 6.9	83.5 +/− 6.7	81.7 +/− 7.0	NS
BMI	25.4 +/− 4.1	24.9 +/− 4,2	24.9 +/− 4.1	NS
Coronary artery disease	70 (46.6 %)	32 (41.0 %)	38 (52.8 %)	NS
PCI	43 (28.7 %)	21 (27.0 %)	22 (30.6 %)	NS
Previous myocardial infarction	16 (10.7 %)	7 (9.0 %)	9 (12.5 %)	NS
Previous cardiac surgery	31 (20.7 %)	12 (15.4 %)	19 (26.4 %)	0.096
CABG	28 (18.7 %)	10 (12.8 %)	18 (25.0 %)	0.056
Mitral mechanical prosthesis	1 (0.7 %)	0 (0 %)	1 (1.4 %)	NS
Aortic bioprosthesis	2 (1.3 %)	2 (2.6 %)	0 (0 %)	NS
Aortic balloon valvuloplasty	3 (2 %)	1 (1.3 %)	2 (2.8 %)	NS
Hypertension	89 (59.3 %)	47 (60.3 %)	42 (58.4 %)	NS
Diabetes	42 (28 %)	24 (30.8 %)	18 (25.0 %)	NS
Smoking	46 (30.6 %)	17 (21.8 %)	29 (40.3 %)	0.014
Plasma creatinine (μmol/L)	113.5 +/−115.2	113.4+/−127.6	114.0 +/−101.2	NS
Renal dialysis	3 (2 %)	2 (2.6 %)	1 (1.4 %)	NS
COPD	24 (16 %)	8 (10.3 %)	16 (22.2 %)	0.046
Peripheral vascular disease	25 (16.6 %)	11 (14.1 %)	14 (19.4 %)	NS
Cerebrovascular disease	20 (13.3 %)	12 (15.4 %)	8 (11.1 %)	NS
Euroscore	21.67 +/− 11.3	20.5 +/− 10.4	22.9 +/− 12.1	NS
STS	9.65 +/− 5.95	8.8 +/− 4.5	10.3 +/− 7.0	NS
Acute pulmonary edema	21 (14 %)	6 (7.7 %)	15 (20.8 %)	0.020
Heart failure	39 (26 %)	15 (19.2 %)	24 (33.3 %)	0.049
Syncope	6 (4 %)	3 (3.8 %)	3 (41.7 %)	NS
Angor pectoris	11 (7.4 %)	7 (9.0 %)	4 (5.6 %)	NS

The primary differences were the following: there were more patients with a history of heart and coronary bypass surgery in the apical group (*p* = 0.096 and *p* = 0.056, respectively); there were significantly more smokers and cases of chronic obstructive pulmonary disease (COPD) in the apical group (*p* = 0.014 and *p* = 0.046, respectively). In addition, with regard to symptomatology, there were significantly more patients presenting with acute pulmonary edema and cardiac decompensation in the apical group (*p* = 0.02 and *p* = 0.049, respectively).

The echographic features are presented in Table 2, the mean gradient and aortic orifice area were 52.8 mmHg and 0.6 cm^2^. Mean left ventricular ejection fraction (LVEF) was 52.8 %, 49.3 % of patients exhibited associated aortic insufficiency, 42 % had associated mitral regurgitation, and the mean systolic arterial pressure was 46.3 mmHg.

**Table 2:** Echocardiographic characteristics at baseline

Echocardiographic characteristics	All patients (*n* = 150)	Transfemoral approach (*n* = 78)	Transapical approach (*n* = 72)	*p*
Mean gradient (mmHg)	52.8 +/− 11.5	52.8 +/− 11.2	52.8 +/− 11.8	0.626
Maximal gradient (mmHg)	83.7 +/− 16.1	85.2 +/− 15.7	82.2 +/− 16.5	0.064
Vmax (m/s)	4.3 +/− 0.8	4.6 +/− 0.7	4.7 +/− 0.7	0.678
Aortic area (cm^2^)	0.6 +/− 0.2	0.6 +/− 0.1	0.6 +/− 0.2	0.778
LVEF (%)	52.8 +/− 12.5	51.3 +/− 13.1	54.5 +/− 11.7	0.140
Diastolic function E/E’	11.4 +/− 4.2	12.0 +/− 4.7	10.9 +/− 3.5	0.115
Aortic annulus diameter (mm)	22.4 +/− 2.7	22.1 +/− 3.1	22.8 +/− 2.2	0.089
IVS (mm)	12.4+/− 3.0	12.6 +/− 2.4	12.7 +/− 2.3	0.936
Aortic regurgitation	74 (49.3 %)	36 (46.2 %)	38 (52.8 %)	0.137
Grade 1	38 (26 %)	22 (28.2 %)	17 (23.6 %)	NS
Grade 2	34 (22 %)	12 (15.3 %)	21 (29.2 %)	NS
Grade 3	2 (1.3 %)	2 (2.6 %)	0 (%)	NS
Mitral regurgitation	63 (42 %)	27 (34.6 %)	36 (50.0 %)	0.128
Grade 1	29 (19.3 %)	12 (15.4 %)	17 (23.6 %)	NS
Grade 2	31 (20.7 %)	15 (19.2 %)	16 (22.2 %)	NS
Grade 3	3 (2 %)	0 (0 %)	3 (4.2 %)	NS
PASP (mmHg)	46.3 +/− 12.6	47.6 +/− 13.0	44.7 +/− 12.0	0.147
TAPSE (mm)	15 +/− 3	14.6 +/− 3.2	15.8 +/− 2.8	0.013
Bicuspid aortic valve	1 (0.7 %)	0 (0 %)	1 (1.4 %)	NS

*IVS* interventricular septum, *LVEF* left ventricular ejection fraction, *PASP* pulmonary artery systolic pressure, *TAPSE* tricuspid annular plane systolic excursion

**Table 3:** Implanted prostheses diameters

Prosthesis diameter (mm)	All prostheses implanted (*n* = 149)	Transfemoral approach (*n* = 78)	Transapical approach (*n* = 72)	*p*
23	52 (34.9 %)	30 (38.5 %)	22 (30.6 %)	0.169
26	64 (42.9 %)	37 (47.4 %)	27 (37.5 %)	0.154
29	25 (16.8 %)	6 (7.7 %)	21 (29.2 %)	<0.001
31	6 (4.0 %)	5 (6.4 %)	1 (1.4 %)	0.083

### Perioperative results

A total of 78 patients were treated using the femoral route and 72 by the apical route. All operations were carried out under general anesthetic. Only 149 prostheses were implanted, as one patient died when anesthetized. Of all the prostheses, 137 were Edwards Sapien and 12 were CoreValve. The mean prosthesis diameter was 25.6 mm. The success rate for the procedure was 94.6 %, taking into account the six patients who died immediately during the operation and two failed procedures. The different reasons for death in the operating theatre were: one massive post-dilation aortic insufficiency, one cardiac arrest under anaesthetic, two ruptures of the aortic root after insertion of the prosthesis, and two cases of cardiac tamponade with refractory state of shock. The two failed procedure were one incorrect position of the valve because of undersizing of the prosthesis resulting in the migration of the valve into the left ventricle, treated by surgical conversion and aortic replacement, and one cardiac tamponade after insertion of the catheter via the apical approach. The mean length of hospital stay was 12.8 days +/− 10.7 days. Comparing the two approach routes, the apical group presented a significantly longer mean hospital stay length, at 15.5 days +/− 12.8 versus 10.3 days +/− 7.6 for the femoral group (*p* = 0.002).

### Mortality rates

**Fig. 1:**
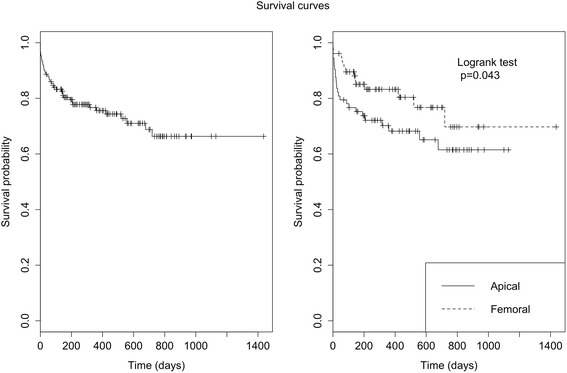
Presents the probability of survival in both the whole population and the population who died from cardiovascular diseases. Day 0 corresponds to the day of the TAVI procedure. We have not lost any of the patients to follow up. Survival rates reported at D1400 correspond to the retrospective time of the last included patient

**Fig. 2:**
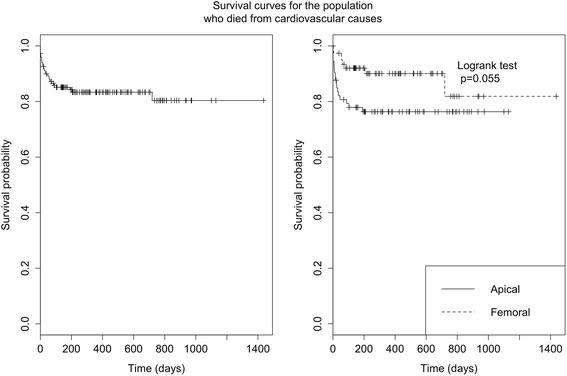
Compares mortality according to route of approach, with the overall mortality rate for the femoral route and the apical route at 14 % and 25 % at 6 months, 18 % and 30 % at 1 year, and 22 % and 39 % at 2 years, respectively

In total, 39 patients died. Our study found that six died in the operating theatre (4 %), 11 later within 31 days (11.3 %), 14 within six months (21.3 %), two at one year (22.7 %), and a further six had died by the 2-year point (26 %). There were no further deaths after 2 years of follow up and the mortality rate at both three and nine months was therefore 26 %. By the 2-year follow-up, 25 patients had died from cardiovascular causes (16.7 %) and 14 patients from non-cardiovascular causes (9.3 %). The different causes of mortality were: dislocation of the shoulder followed by confinement to bed, hepatocellular cancer, septic shock passing a kidney stone, colon cancer, two cases of postoperative failure to thrive syndrome, kidney failure after 5 months, hepatocellular failure with cirrhosis, confinement to the postoperative cerebrovascular accident (CVA) suite, rectorrhagia, cerebral lymphoma, and three unexplained causes at one year. Note that one patient died suddenly at home six months after discharge from the hospital, the diagnosis of sudden death was made; the patient did not have a pacemaker (Figs. 1 and 2).

In our study, the probability of overall survival was higher for the femoral approach group than the apical group by a significant degree (*p* = 0.043). On comparing cardiovascular mortality according to the route of approach, the rates were 10 % vs. 21 % for the femoral and apical routes, respectively, at 6 months, 10 % vs. 21 % at 1 year, and 19 % vs. 25 % at 2 years. We found a non-significant trend towards an increased probability of survival in the femoral group compared to the apical group (*p* = 0.055).

### Predictive factors of mortality

We found few predictive factors of cardiovascular mortality. In particular, the Euroscore and STS surgical risk assessment scores were not found to be significant. By analyzing single variables, we found the following predictive factors for cardiovascular mortality: preoperative mitral regurgitation ≥ Grade 2 (*p* = 0.024) and survival of major complications (=0.001). However, a tendency towards an increase in the cardiovascular mortality rate was found in the following contexts: where coronary disease was present (*p* = 0.07), in females (*p* = 0.053), with NYHA Class II or IV dyspnoea (*p* = 0.09), and with the apical approach (*p* = 0.08). On multivariate analysis, only the presence of a major complication was significantly predictive of cardiovascular mortality (*p* = 0.003). Detailed results are presented in Table 4.

**Table 4:** Predictive factors of mortality (multivariate analysis)

Characteristics	RR	95 % CI	*p*
Female gender	2.45	[0.99–6.03]	0.051
Coronaropathy	1.84	[0.76–4.49]	0.177
NYHA Class 3 or 4	3.92	[0.52–29.6]	0.185
Major complications	9.13	[2.12–39.4]	0.003
Transapical approach	1.62	[0.7–3.75]	0.258
Mitral regurgitation (≥ Grade 2)	1.81	[0.8–4.22]	0.166

*RR* relative risk, *CI* confidence interval, *NYHA* New York Heart Association

### Complications

The postoperative complications of the whole population have been classified and compared according to route of approach, presented in Table 5.

**Table 5:** Postoperative complications

Characteristics	All patients (*n* = 150)	Transfemoral approach (*n* = 78)	Transapical approach (*n* = 72)	*p*
Hemodynamic complications	10 (6.6 %)	2 (2.6 %)	8 (11.1 %)	0.049
state of cardiogenic shock	4 (2.7 %)	0 (0 %)	4 (5.6 %)	0.035
acute pulmonary edema	5 (3.4 %)	1 (1.3 %)	4 (5.6 %)	NSS
multiple organ failure	9 (6 %)	2 (2.6 %)	7 (9.7 %)	0.065
coronary obstruction	1 (0.7 %)	1 (1.3 %)	0 (0 %)	NSS
massive aortic insufficiency	1 (0.7 %)	0 (0 %)	1 (1.4 %)	NSS
Vascular complications	20 (13.3 %)	11 (14.1 %)	9 (12.5 %)	NSS
major vascular complications	7 (4.7 %)	4 (2.7 %)	3 (4.2 %)	NSS
minor vascular complications	2 (1.3 %)	1 (1.3 %)	1 (1.4 %)	NSS
mesenteric ischemia	1 (0.7 %)	1 (1.3 %)	0 (0 %)	NSS
abdominal aortic aneurysm	1 (0.7 %)	1 (1.3 %)	0 (0 %)	NSS
Tamponade	11 (7.4 %)	7 (9.0 %)	4 (5.6 %)	NSS
Mechanical complications	8 (5.3 %)	2 (2.6 %)	6 (8.3 %)	NSS
ventricular perforation	2 (1.3 %)	0 (0 %)	2 (2.8 %)	NSS
VSD	1 (0.7 %)	1 (1.3 %)	0 (0 %)	NSS
aortic root aneurysm	1 (0.7 %)	0 (0 %)	1 (1.4 %)	NSS
aortic fistula/right atrium	1 (0.7 %)	1 (1.3 %)	0 (0 %)	NSS
mitral lesion	1 (0.7 %)	0 (0 %)	1 (1.4 %)	NSS
poor positioning of the valve	2 (1.3 %)	0 (0 %)	2 (2.8 %)	NSS
surgical conversion	3 (2 %)	0 (0 %)	3 (4.2 %)	NSS
Hemorrhagic complications	55 (36.7 %)	20 (25.7 %)	33 (45.8 %)	NSS
major complications	10 (6.7 %)	3 (3.8 %)	7 (9.7 %)	NSS
massive bleeding	1 (0.7 %)	0 (0 %)	1 (1.4 %)	NSS
major bleeding	7 (4.7 %)	3 (3.8 %)	4 (5.6 %)	NSS
minor bleeding	36 (24 %)	14 (18.0 %)	22 (30.6 %)	0.070
transfusion	42 (28 %)	15 (19.2 %)	27 (37.5 %)	0.013
Cerebral complications	10 (6.7 %)	4 (5.1 %)	6 (10.0 %)	NSS
Ischemic CVA	9 (6 %)	4 (5.1 %)	5 (7.0 %)	NSS
Hemorrhagic CVA	1 (0.7 %)	0 (0 %)	1 (1.4 %)	NSS
Heart rhythm complications				
paroxysmal atrial fibrillation	24 (16 %)	13 (16.7.7 %)	11 (15.3 %)	NSS
pacemaker implanted	18 (12 %)	12 (15.4 %)	6 (8.3 %)	NSS
sudden death	1 (0.7 %)	0 (0 %)	1 (1.4 %)	NSS
Acute kidney injury (AKI)	15 (10 %)	7 % (9.0 %)	9 (12.5 %)	NSS
Stage 1	2 (1.3 %)	1 (1.3 %)	1 (1.4 %)	NSS
Stage 2	7 (4.7 %)	3 (3.8 %)	4 (4.2 %)	NSS
Stage 3	6 (4 %)	3 (3.8 %)	3 (2 %)	NSS
Pulmonary complications	14 (9.3 %)	2 (2.6 %)	12 (8 %)	0.029
pleural effusion	5 (3.3 %)	1 (1.3 %)	4 (5.6 %)	NSS
hemothorax	1 (0.7 %)	0 (0 %)	1 (0.7 %)	NSS
pneumothorax	3 (2 %)	0 (0 %)	3 (4.2 %)	NSS
Infectious complications	18 (12 %)	12 (15.4 %)	6 (8.3 %)	NSS
Scarpa’s fascia infection	11 (7.4 %)	10 (12.8 %)	1 (1.4 %)	0.007
thoracotomy infection	4 (2.7 %)	0 (0 %)	4 (5.6 %)	0.048
endocarditis	3 (2 %)	2 (2.6 %)	1 (1.4 %)	NSS

*CVA* cerebrovascular accident, *VSA* ventricular septal defect, *AKI* Acute kidney injury

A comparison of the rates of reported complications in terms of approach method revealed significantly more hemodynamic complications among the apical group (*p* = 0.049). None of the patients who underwent surgery via the femoral route presented with a postoperative state of cardiogenic shock (*p* = 0.035). Seven patients who had surgery via the apical route presented with multiple organ failure, compared to two of those operated via the femoral route (*p* = 0.065).

On the other hand, we noted an insignificant difference in the level of minor bleeding (*p* = 0.07) for the femoral group. Transfusions were significantly more frequent in the apical approach group, with 18 % for the femoral approach versus 28 % for the apical approach (*p* = 0.013). Pulmonary complications were also significantly more frequent in the apical approach cases (*p* = 0.029). As regards infections, the rate of Scarpa’s fascia infection was significantly higher in cases using the femoral approach (*p* = 0.007) and thoracotomy infections were significantly higher for the apical approach (*p* = 0.048). Otherwise, a total of 18 pacemakers were fitted, 12/135 Edwards Sapien valves and 6/12 CoreValves. Finally, concerning the levels of renal insufficiency, these were divided into 3 stages depending on the severity of the case, using VARC classification (Stage 1 for an increase in creatininemia of 150 to 199 %, stage 2 for an increase of 200 % to 299 %, and stage 3 for an increase of more than 300 % or anuria for more than 12 h).

### Follow-up

Patients exhibited significant improvement in dyspnea at 1 month (*p* < 0.001), this improvement still proved stable over time. The improvement in functional status was spectacular and there has been a clear improvement in the quality of life of our patients from the first month following the implantation, with over half presenting as NYHA Class I or II.

#### Echocardiographic results

The efficacy of TAVI was confirmed by this investigation, resulting in a drop in mean gradient of hemodynamic flow from 52.8 to 11.8 mmHg (*p* < 0.001), and the aortic surface increased from 0.6 to 1.6 cm^2^ (*p* < 0.001). These parameters remained stable throughout the follow-up period. The interindividual variability of the LVEF improved significantly over time (*p* = 0.001). Postoperative LVEF was 52.4 % on average, compared to 52.8 % prior to surgery. We observed a significant improvement in the ejection fraction at one month, increasing from 52.4 % to 54.6 % (*p* < 0.026), with this improvement proving stable over time.

There was no significant change in the diastolic function.

The systolic arterial pressure measurements significantly decreased from 46.3 to 40.8 mmHg (*p* < 0.001) and remained stable over time. Detailed results are presented in Table 6.

**Table 6:** Echocardiographic postoperative results

Echocardiographic characteristics	Preoperative	Postoperative	One month	Six months	One year	Two years
Population (*n*)	*n* = 150	*n* = 144	*n* = 125	*n* = 101	*n* = 52	*n* = 25
Mean gradient (mmHg)	52.8 +/−11.5	11.8+/−4.9	11+/−4.6	11.5+/−5.2	11.9+/−4.4	11.2+/−3.8
*p* < 0.001		*p* < 0.001				
Maximal gradient (mmHg)	83.7 +/−16.1	21.5+/−8.1	22.1+/−18.8	21.4+/−7.7	20.5+/−5.6	19.6+/−4.3
*p* < 0.001		*p* < 0.001				
Vmax (m/s)	4.3 +/−0.8	2.1+/−0.4	1.7+/−0.5	1.7+/−0.6	1.6+/−0.5	1.7+/−0.5
*p* < 0.001		*p* < 0.001				
Aortic area (cm^2^)	0.6 +/−0.2	1.6+/−0.4	1.2+/−0.4	1.2+/−0.3	1.2+/−0.4	1.2+/−0.4
*p* < 0.001		*p* < 0.001				
LVEF (%)	52.8+/−12.5	52.4+/−12.1	54.6+/−10.4	55+/−10.7	54.4+/−10.9	52.8+/−12.5
*p* = 0.002			*p* = 0.026	*p* = 0.056		
Diastolic function E/E’	11.4+/−4.2	11.4+/−3.8	11.5+/−3.4	11.7+/−3.8	11.5+/−3.4	11.7+/−3.9
NS						
PAPS (mmHg)	46.3+/− 12.6	40.8+/−12	41.7+/−10.7	41.5+/−10.5	40.5+/−11.2	38.8+/−6.4
*p* = 0.006		*p* < 0.001	*p* = 0.078	*p* = 0.045		
TAPSE (mm)	15.2+/−3	13.8+/−3.2	15.4+/−3	15.1+/−2.9	14.8+/−3.4	14.2+/−3.2
NS						
Central aortic regurgitation	/	4 (2.8 %)	3 (2.4 %)	2 (2.0 %)	1 (1.9 %)	0 (0 %)
NS						
Paraprosthetic regurgitation	/	74 (51.4 %)	38 (30.4 %)	14 (13.8 %)	10 (19.2 %)	8 (32.0 %)
*p* < 0.001						
Grade 1	/	35 (24.3 %)	24 (19.2 %)	6 (59.4 %)	6 (11.5 %)	6 (24.0 %)
Grade 2	/	39 (27.1 %)	13 (10.6 %)	8 (7.9 %)	4 (7.6 %)	2 (8 %)
Mitral regurgitation	63 (42 %)	83 (57.6 %)	88 (70.6 %)	72 (71.2 %)	37 (71.1 %)	19 (76.0 %)
Grade 1	29 (19.3 %)	12 (8.3 %)	49 (32.2 %)	33 (32.6 %)	15 (19.2 %)	8 (32.0 %)
Grade 2	31 (20.7 %)	71 (49.3 %)	39 (31.2 %)	39 (38.6 %)	22 (42.3 %)	11 (44.0 %)
Grade 3	3 (2 %)	0 (0 %)	0 (0 %)	0 (0 %)	0 (0 %)	0 (0 %)

*LVEF* left ventricular ejection fraction, *PASP* pulmonary artery systolic pressure, *TAPSE* tricuspid annular plane systolic excursion

## Discussion

The procedure success rate, defined as the correct deployment of the prosthesis, was 94.6 % in our study, in line with that of series reporting success rates with this procedure of over 90 % at the test centers.[8, 9] As we have detailed, we have kept the 6 patients who died in the operating theatre within the 5.4 % failure rate. Among the causes of implantation failure, one death was linked to the anaesthesia, a secondary one to aortic predilation, another secondary to inserting the catheter using the apical approach and the other causes were secondary to deployment of the prosthesis.

Otherwise, with regard to aortic leaks, it is currently established thinking that a paraprosthetic leak ≥ Grade 2 increases mortality from the 6^th^ month following implantation [5, 10]. Our study reported accurate results, with a rate of 49.3 % for minor or moderate postoperative paraprosthetic leaks (35 % at Grade 1 and 39 % at Grade 2). By means of a comparison, we also searched the FRANCE 2 data, revealing 64.5 % aortic failure [5]. Moreover, aortic regurgitation remains a challenging pathology for the transapical approach [11]. Concerning the evolution of paraprosthetic aortic insufficiency, our study indeed found a tendency towards increasing the percentage of aortic insufficiency at 6 months, 1 year, then 2 years. Apart from statistical bias linked to the smaller population over time, this may be linked to a general cardiovascular aggravation, with a possible increase in left ventricular postload. An important point is the lack of major aortic insufficiency.

A large proportion of our study consisted of TAVI carried out using the apical approach, with 77 of the patients (51.3 %) treated using the femoral route and 72 (48 %) using the apical route. The usual distribution is more in favor of the femoral approach: 74 % of the patients in FRANCE 2 [5] and 69.5 % in PARTNER A [3] received the implant via the femoral route, compared with 19 % and 29.6 %, respectively, for the apical route. This significantly higher percentage for the apical route is due in part to the delay in marketing the Edwards 29 valve for the femoral route. Mortality at 6 months essentially results from extracardiac causes and is linked to comorbidities in elderly patients. This difference in mortality rates is probably explained by the increased use of the apical approach among our population, with a trend of increased mortality in this cohort. It has, in fact, been demonstrated that mortality rates are more significant in the apical approach than in the femoral one [12–15].

Patients treated via the transapical route are typically at higher risk. To date, there have been few studies comparing different devices or approaches. It should, nevertheless, be noted that all reports have indicated a learning curve effect on the success rate, incidence rate, and severity of the complications. Hemodynamic complications are a major cause of perioperative death [16]. These represent 24 % of deaths at the one month mark [16]. The significant difference in hemodynamic complications found in our study, greater in the femoral group (Table 3), also explains the difference in mortality rate between the two groups.

The rate of acute kidney injury (AKI) in our study (10 %) were slightly lower than those reported in the literature.[17–19] Post-TAVI AKI was multifactorial, since preoperative renal function is a predictive factor independent of post-procedural AKI [19]. Note that the level of plasma creatinine in our cohort was 113.5 μmol/L +/−115.2, and of the six patients who suffered stage 3 AKI, none of them underwent dialysis apart from 3 patients who were receiving dialysis for a chronic condition already.

The rate of pulmonary complications was higher in the apical group compared with the femoral group, at 8 % vs. 1.3 % (*p* = 0.029). This constituted one of the most predominant causes of morbidity and mortality in the apical group. Nevertheless, we found no significant difference in rate of tamponade during the postoperative period when comparing the two approaches, namely 4.7 % in the apical group versus 2.7 % in the femoral group.

Pre-TAVI mitral regurgitation was identified in our study as a risk factor for mortality when Grade 2 or higher, as other studies have also observed [20–23]. The post-TAVI mitral regurgitation in our cohort showed a tendency to increase over time. The evolution of post-TAVI mitral regurgitation remains discordant, depending on the studies [3, 24, 25]. In our case, it can be explained by statistical bias linked to a reduction in population monitored over time, general age-linked cardiovascular deterioration, the tendency towards an increase in aortic insufficiency in our cohort or the high proportion of ischemic patients (46.6 %).On the other hand, the mechanism underlying the mitral regurgitation is an important factor [26]. The functional nature of the leak and presence of left ventricular failure are predictive factors for the reduction in mitral valve disease. Conversely, its organic nature, the dilatation of the left atrium, and the existence of pulmonary artery hypertension all suggest a lack of improvement [27].

Finally, the rate of major vascular complications was relatively low in our study, reported at 4.7 %. Taking into account minor vascular complications, the overall rate was 6 %. This could be as a result of the high percentage of apical approaches used. Vascular complications were found to remain a significant source of morbidity in the transfemoral route, with an incidence of 9.7 % [5]. Reduction in major vascular complications from 8 to 1 % has been demonstrated with the benefit of a more precise selection of patients, a completely percutaneous vascular approach, and advances in surgical techniques [28]. This demonstrated that the number of vascular complications and survival rate increases in parallel with increased experience of each center and over time [29].

## Conclusions

TAVI has been confirmed as a credible alternative to surgical valve replacement and has become the first therapeutic choice for non-operable patients and a valid alternative for high-risk patients. The mid-term results of the first 150 TAVI procedures, conducted from 2009 to 2013 in our center, demonstrated mortality rates of 4 %, 11.3 %, 22.7 % and 26 % at the immediate perioperative point, 31 days, 1 year, and 2 years, respectively. The interindividual variability of LVEF improved significantly over time (*p* = 0.001). Our study revealed a trend towards increased probability of survival in the femoral group compared to the apical group. On comparing the rates of complications in terms of approach method, we observed that the patients treated through the transapical route, who are usually at higher risk, exhibited significantly more hemodynamic complications (*p* = 0.049) and more pulmonary complications (*p* = 0.029). These results underline the importance of a multidisciplinary decision concerning the choice of approach type.

## Abbreviations

AKI: acute kidney injury
CI: confidence interval
IQR: interquartile range
OR: odds ratio
STS: The Society of Thoracic Surgeons
TA-AVI: transapical aortic valve implantation
TAVI: transcatheter aortic valve implantation
TF-AVI: transfemoral aortic valve implantation
VARC: Valve Academic Research Consortium
